# Impact of Temperature on the Immune Interaction between a Parasitoid Wasp and *Drosophila* Host Species

**DOI:** 10.3390/insects12070647

**Published:** 2021-07-15

**Authors:** Fanny Cavigliasso, Jean-Luc Gatti, Dominique Colinet, Marylène Poirié

**Affiliations:** Université Côte d’Azur, INRAE, CNRS, ISA, 06 903 Sophia Antipolis, France; fanny.cavigliasso@unil.ch (F.C.); jean-luc.gatti@inrae.fr (J.-L.G.); dominique.colinet@inrae.fr (D.C.)

**Keywords:** parasitoid wasp, *Drosophila*, temperature, encapsulation, venom composition, parasitic success, phenotypic plasticity

## Abstract

**Simple Summary:**

Global warming affects most species and their interaction s. Insects are ectotherms, meaning their body temperature is affected by the ambient temperature. This is particularly important for koinobiont parasitoids, insects that keep their host insect alive during development of their eggs and larvae, the host eventually being consumed before adult parasitoids emerge. Temperature changes could therefore affect parasitoids directly and/or indirectly through their impact on the host. Here, we tested the effect of temperature on the parasitic success of two parasitoid lines on two host species, and on each partner independently, to determine whether the host immune response and/or the parasitoid venom proteins, injected with the egg to counteract the host immune response, were affected. The host’s immune defense consists of forming a capsule surrounding the parasitoid egg. In half of the interactions tested, the parasitic success increased with temperature. For one, the increase appeared to result solely from an increased capacity of the parasitoid to escape from a capsule, while for the second, it also appeared to involve a decrease in host encapsulation capacity. Finally, we observed a strong change in venom composition depending on the rearing temperature which may partially explain the change in parasitic success.

**Abstract:**

Temperature is particularly important for ectotherms, including endoparasitoid wasps that develop inside another ectotherm host. In this study, we tested the impact of three temperatures (20 °C, 25 °C and 30 °C) on the host–parasitoid immune interaction using two *Drosophila* host species (*Drosophila melanogaster* and *D. yakuba*) and two parasitoid lines of *Leptopilina boulardi*. *Drosophila*’s immune defense against parasitoids consists of the formation of a melanized capsule surrounding the parasitoid egg. To counteract this response, *Leptopilina* parasitoids rely on the injection of venom during oviposition. Here, we tested the effect of temperature on parasitic success and host encapsulation capacity in response to a parasitoid egg or other foreign body. Increased temperature either promoted or did not affect the parasitic success, depending on the parasitoid–host pairs considered. The mechanisms behind the higher success seemed to vary depending on whether the temperature primarily affected the host immune response or also affected the parasitoid counter-immune response. Next, we tested the effect of parasitoid rearing temperature on its success and venom composition. Venom composition varied strongly with temperature for both parasitoid lines, partially consistent with a change in their parasitic success. Overall, temperature may have a significant impact on the host–parasitoid immune interaction.

## 1. Introduction

Due to global warming, the average temperature of Earth’s surface is projected to increase by 1.0 to 4.1 °C during the 21st century [1]. This temperature change is expected to significantly affect species and their interactions [2,3,4]. As insects are ectothermic organisms, their physiological processes and life-history traits are particularly sensitive to temperature, as reported, for example, for metabolism and respiration [5], developmental time [6], induction of diapause [7] and body size [8]. Among insects, the impact of climate change is expected to be particularly important for parasitoids because they develop at the expense of other ectotherms, usually insects. This is especially true for koinobiont parasitoids that require the host to remain alive during most of the parasitoid’s larval development [9]. Temperature can therefore affect koinobiont parasitoids, not only directly, but also indirectly, through its impact on host physiology and/or immunity. Because of the ecological role of parasitoids in controlling insect populations and their use as biological control auxiliaries, studies have focused on the consequences of temperature changes on host–parasitoid interactions in the context of global warming (see for example: [10,11,12,13,14,15]).

In many of the host–parasitoid interactions studied, the parasitoid was negatively affected by increased temperature and a decrease in parasitic success was observed [16,17,18,19,20,21,22]. However, although less frequent, cases where high temperatures favored the parasitoid over the host were also observed [6,23,24,25]. Furthermore, although the success of parasitism depends notably on the ability of the host to mount an immune response against the parasitoid and the ability of the parasitoid to avoid or counteract this response, the impact of temperature on these processes has rarely been tested for each partner independently. To our knowledge, only a few studies have investigated the impact of temperature on the host immune response independently of the parasitoid. In these studies, the deposit of a parasitoid egg was simulated with cactus thorns [26], nylon filaments [27] or Sephadex^TM^ beads [28].

The possible indirect effect of temperature on parasitoids is illustrated by a recent study [28] involving a parasitoid of the super-family Ichneumonoidea that produces viral particles (Polydnaviruses) containing DNA circles that notably encode virulence genes. The parasitoid injects these particles into the host together with the eggs and they enter its cells, the virulence genes being therefore expressed inside host cells. At high temperature, the authors describe a downregulation of the transcription of several of these genes involved in parasitic success. The observed decrease in the level of transcription therefore depends on the impact of temperature on the host but not on the parasitoid. The situation is clearly different with *Leptopilina boulardi*—the parasitoid used in this study—which does not produce viral particles but venom proteins, circulating or enclosed in vesicles produced in the venom apparatus. In this case, no indirect effect of temperature via the host could affect the amount of virulence proteins delivered upon parasitism.

The objective of our work was to study the impact of temperature on the outcome of the host–parasitoid interaction, as well as on each partner independently, to investigate the likely mechanisms between host encapsulation and/or parasitoid venom composition. To address these questions, we used the *Drosophila-Leptopilina boulardi* interaction model because the mechanisms involved in the *Drosophila* immune defense and in the ability of *L. boulardi* to overcome this response have been thoroughly investigated [29,30]. *L. boulardi* is a koinobiont endoparasitoid whose females inject one or more eggs into *Drosophila* larvae, with only one egg developing to an adult parasitoid in case of parasitic success. The *Drosophila* immune defense consists of a process called encapsulation which involves the deposit of several layers of hemocytes around the parasitoid egg and the parallel activation of the phenoloxidase cascade which leads to the production of melanin and reactive radicals species (ROS and RNS) that are believed to play a major role in parasitoid death [31,32,33]. The strategy by which *L. boulardi* counteracts the *Drosophila* immune response is primarily the injection of venom proteins by the female parasitoid during oviposition [29,30]. An interesting aspect of the model is that the impact of temperature on the encapsulation process and on the protein composition of the parasitoid venom can be analyzed independently. Oviposition by *L. boulardi* females can be simulated by injecting oil drops into *Drosophila* larvae to assess the host’s ability to form a melanized capsule against a large foreign body [34,35]. In addition, the protein composition of venom from *L. boulardi* females can be analyzed and compared at the individual level [36,37,38,39].

Another interesting feature of the model is the existence of two well-characterized lines of *L. boulardi*, named ISm and ISy, which differ in their virulence properties and venom composition [29,40]. The success of the ISm line is host species-dependent, as it always succeeds on *D. melanogaster* but is consistently encapsulated by *D. yakuba*. The ISy line can succeed on both *Drosophila* species depending on the resistance/susceptibility of the host strains [34,41]. The venom of these lines differs widely, primarily due to quantitative differences in venom proteins. For example, LbGAP, a RhoGAP domain-containing protein, is much more abundant in ISm venom than in ISy [42]. This protein appears to be necessary for the parasitic success of ISm on a particular strain of *D. melanogaster* that is resistant to ISy, targeting the host immune cells dedicated to encapsulation [42,43,44,45,46]. Qualitative variation in venom proteins between ISm and ISy was also demonstrated. For example, differences between the two lines in the active site of LbSPN, a serine protease inhibitor of the serpin superfamily, suggest different targets and/or functions for this abundant venom protein [29,47]. While LbSPN in ISy (called LbSPNy) is known to target the phenoloxidase cascade—a crucial step for the melanization process—in *D. yakuba* [47], its role in ISm (called LbSPNm) is yet to be evidenced.

Here, we report data on the plastic responses to temperature of: (i) four interaction outcomes (parasitism rate, parasitic success, host encapsulation capacity and parasitoid ability to escape from a capsule) between each of two *Drosophila* species (*D. melanogaster* and *D. yakuba*) and two lines of *L. boulardi* (ISm and ISy); (ii) host encapsulation capacity by simulating foreign body injection (through paraffin oil injection); (iii) parasitic success and venom composition of the two parasitoid lines of *L. boulardi*. The temperatures tested were 25 °C, the laboratory rearing temperature at which *L. boulardi* does not diapause [7], and 20 °C and 30 °C, easily observed under natural conditions but inducing a lower development rate in both partners [48,49,50].

## 2. Materials and Methods

### 2.1. Biological Material

The isofemale lines ISm (Gif stock number 431) and ISy (Gif stock number 486) *of L. boulardi* originate from populations collected in Nasrallah (Tunisia) and Brazzaville (Congo), respectively [51]. Both lines were reared at 25 °C on their susceptible maintenance strain of *D. melanogaster* (Nasrallah from Tunisia, Gif stock number 1333, here named SNasr). Emerged adults were maintained at 20 °C on agar medium with honey.

Two *Drosophila* host strains from two different species differing in their resistant/susceptible phenotype to ISm and ISy were used. The *D. melanogaster* R strain (Gif stock number 1088, referred to here as *D. melanogaster*) was derived from isofemale lines obtained from a population of Brazzaville, Congo [52] by subsequent genetic approaches [51,52]. It is resistant to the ISy line of *L. boulardi* and susceptible to the ISm line [51,53]. Conversely, *Drosophila yakuba* 307 strain (Gif stock number 307.14, here named *D. yakuba*) from a population collected in 2004 in Annobon, Sao Tome, is resistant to ISm and susceptible to ISy.

### 2.2. Samples Preparation for Identification of the Developmental Stage at Which Venom Synthesis Begins

The *D. melanogaster* S_Nasr_ strain (approximately one week old; susceptible to both lines of *L. boulardi*) was allowed to lay eggs for 4 h in vials containing nutrient medium. Forty-eight h later, 10 female parasitoids (ISm or ISy) were allowed to parasitize *Drosophila* larvae for 4 h at 25 °C. Between 15 and 22 days post-parasitism, the parasitoid nymph females, recognizable by the length of their antennae, were collected from *Drosophila* pupae. The abdomens of parasitoid females were individually crushed in 15 μL of a solution composed of equivalent volumes of insect Ringer, supplemented with a cocktail of protease inhibitors (PI; Roche), and Laemmli reducing buffer. Samples were then heated (95 °C, 10 min), centrifuged 1 min at 1000× *g*, and the supernatant was stored at −20 °C until Western blot analysis (see Section 2.3 below).

### 2.3. Western Blot Analysis

Proteins from each sample were separated on 1D SDS-PAGE 12.5% gels. The electrophoretic gels were then blotted, according to [54], onto nitrocellulose membranes (120 V, 1 h; Protran B85, GE Healthcare Life Science, Velizy-Villacoublay, France). The membranes were incubated 1 h in 2% milk in TBS-Tween (20 mM Tris-HCl pH 7.3, 150 mM NaCl, 0.2% Tween 20) and overnight at 4 °C in a mix of rabbit polyclonal antibodies directed against LbGAP (1:10,000), LbGAP2 (1:2000) or LbSPN (that recognizes both LbSPNm and LbSPNy) (1:2000) in 2% milk in TBS-Tween. The three polyclonal antibodies are described in [29,43,44,55]. After three washes in TBS-Tween, the membranes were incubated 2 h with a secondary goat anti-rabbit IgG horseradish peroxidase conjugate (1:10,000; Sigma, St. Quentin Fallavier, France) in 2% milk in TBS-Tween, washed three times ins TBS-Tween and revealed with a luminescent substrate (LuminataTM Crescendo Western HRP Substrate; Millipore, Burlington, MA, USA). Digital images of the blots were then acquired with a cooled CCD camera (Andor iKon-M, Abington-on-Thames, UK).

### 2.4. Analysis of the Host-Parasitoid Interaction Outcomes

**Figure 1:**
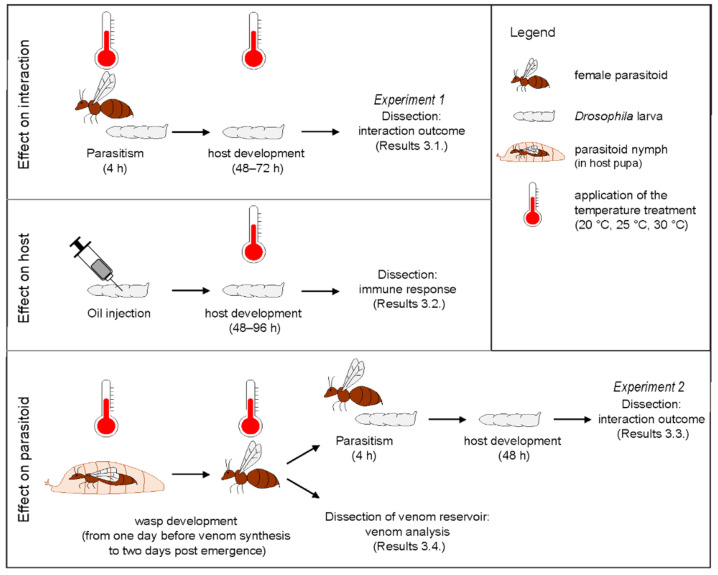
Schematic illustration of the experimental designs. A thermometer indicates where different temperatures (20 °C, 25 °C or 30 °C) were applied, while the absence of a thermometer means that the step was performed at 25 °C.

Two experiments differing in timing and duration of the temperature (Figure 1) were designed to analyze the impact of temperature on host-parasitoid interaction.

For experiment 1 (“effect on interaction” in Figure 1), the temperature treatment was applied during parasitism and the following 48 h, after which parasitized host larvae were dissected. Groups of 30 s-instar larvae of *D. melanogaster* or *D. yakuba* were placed in small dishes containing nutritive medium with one *L. boulardi* ISm or ISy parasitoid female. After 4 h at 20 °C, 25 °C or 30 °C, the parasitoid females were removed, and the dishes were kept at the same temperature until the *Drosophila* larvae were dissected.

For experiment 2 (“effect on parasitoid” in Figure 1), the temperature treatment was applied to developing parasitoids, from one day before the onset of venom synthesis (on days 17 for ISm and 19 for ISy, Supplementary Figure S1) to 48 h after emergence. As in Section 2.2, 10 *L. boulardi* ISm or ISy parasitoid females were allowed to oviposit on 48 h-old larvae of *D. melanogaster* S_Nasr_ at 25 °C. Pupae containing parasitoid nymphs were collected one day before the onset of venom synthesis, divided into three groups and kept on agar medium at 20 °C, 25 °C or 30 °C until parasitoid emergence. Forty-eight h post-emergence, female parasitoids were either used for parasitic tests or stored at −80 °C until dissection of their venom apparatus (Section 2.6 below). For this experiment, 30 early second-instar larvae of *D. melanogaster* or *D. yakuba* were placed in small dishes containing nutritive medium with female parasitoids reared at 20 °C, 25 °C, or 30 °C. The parasitoids were removed after 4 h at 25 °C and the dishes kept at 25 °C until the *Drosophila* larvae were dissected. Because the parasitism rate was very low when the parasitoid nymphs developed at 30 °C, two *L. boulardi* females were used for parasitic tests.

#### 2.4.1. Dissection of Host Larvae

**Figure 2:**
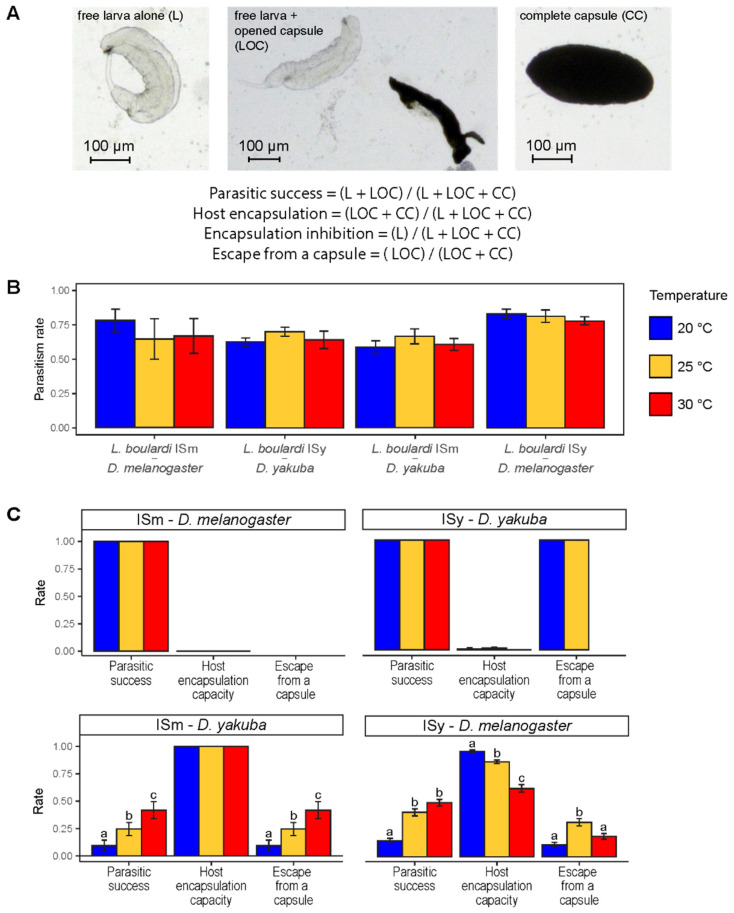
Impact of temperature during parasitism on the outcome of host–parasitoid interaction. (**A**) Possible outcomes in parasitized *Drosophila* larvae 48–72 h after parasitism and details of analyzed parameters: parasitic success, host encapsulation capacity, parasitoid ability to inhibit encapsulation and parasitoid capacity to escape from a capsule. (**B**) Parasitism rates for the four host–parasitoid interactions as a function of temperature. (**C**) Rate of (i) parasitic success, (ii) host encapsulation capacity or (iii) parasitoid capacity to escape from a capsule, depending on the temperature. Within each host–parasitoid interaction, different letters indicate a significant difference between temperatures for a given parameter. Bars indicate standard errors (see Supplementary Tables S1 and S2 for sample sizes and statistical outputs).

*Drosophila* larvae were dissected 48 h after parasitism (or 72 h for dishes in experiment 1 that were kept at 20 °C) and then categorized into (i) non-parasitized, (ii) mono-parasitized and (iii) multi-parasitized. All three categories were used to assess the rate of parasitism (see Section 3.1.), while only mono-parasitized host larvae were considered for the analysis of the interaction outcome to avoid the unpredictable effect of multiple parasitoid larvae in a single host. Three possible outcomes were recorded: (i) free parasitoid larva alone (L), (ii) free parasitoid larva together with an open capsule (LOC) and (iii) complete closed capsule (CC) (Figure 2A). Among these, the percentage of (i) and (iii) has generally been used to assess the immune suppression capacity of the parasitoid and the encapsulation capacity of the host, respectively [56,57,58]. As in [38], outcome (ii) was added to evaluate the ability of the parasitoid to escape the encapsulation process initiated by the host after recognition of the parasitoid egg. Since the escaped parasitoid larva was alive, we considered this outcome as a parasitic success as a free parasitoid.

#### 2.4.2. Statistical Analyses

We fitted generalized linear mixed models (GLMM) with either the parasitism rate (number of parasitized larvae versus number of non-parasitized larvae), the parasitic success (number of larvae with LL or LOC versus number of larvae with CC), the capacity of the host to encapsulate the parasitoid egg (number of larvae with LOC or CC versus number of larvae with LL), the capacity of the parasitoid to inhibit capsule formation (number of larvae with LL versus number of larvae with LOC or CC) or the parasitoid capacity to escape from a capsule (number of larvae with LOC versus number of larvae with CC) as a response variable, with temperature as a fixed factor, replicate (see details below) as a random effect and a binomial error distribution. The biological replicates were of different types between the two experiments. For experiment 1, the temperature treatment was applied during the parasitic test. Therefore, it was possible to perform parasitic tests with all three temperatures at the same time for a given vial containing *Drosophila* larvae of the same age and from the same environment. Therefore, the replicates in experiment 1 correspond to the days on which the parasitic tests were performed. In the case of experiment 2, the development time of parasitoids varies with the rearing temperature, and the parasitic tests were thus performed on different days, depending on the rearing temperature. The replicates in experiment 2 therefore correspond to the different starting vials and not to the days on which the parasitic tests were performed. The GLMM were fitted with the *glmer* function in “lme4” R package [59]. Overdispersion was tested and considered in GLMM when necessary (ratio > 2) by adding a random factor corresponding to the number of observations [60]. Pairwise comparisons with Tukey tests were then performed with *emmeans* and *pairs* functions in R [61]. All statistical outputs and sample sizes are reported in the Supplementary Tables S1–S4.

### 2.5. Analysis of the Host Capacity to Encapsulate an Oil Drop

To avoid the “parasitoid” effect on encapsulation capacity, we simulated egg deposition inside the host by injecting paraffin oil into large second-instar larvae of *D. melanogaster* or *D. yakuba*, using a Nanoject II apparatus (Drummond) and a stretched capillary (Drummond 3-000-203-G/X). The injection was set for an injected volume of 55.2 nl with a slow injection rate. After oil injection, *Drosophila* larvae were transferred to dishes containing medium and placed at 20 °C, 25 °C or 30 °C until the larvae were dissected. Thirty to 40 *Drosophila* larvae were injected per dish and temperature, depending on the number of injections that could be performed with a single capillary. Because the capillaries were prepared manually, the same capillary was used for at least three dishes (i.e., one replicate for each temperature) to reduce variation in the size of the injected oil drop.

**Figure 3:**
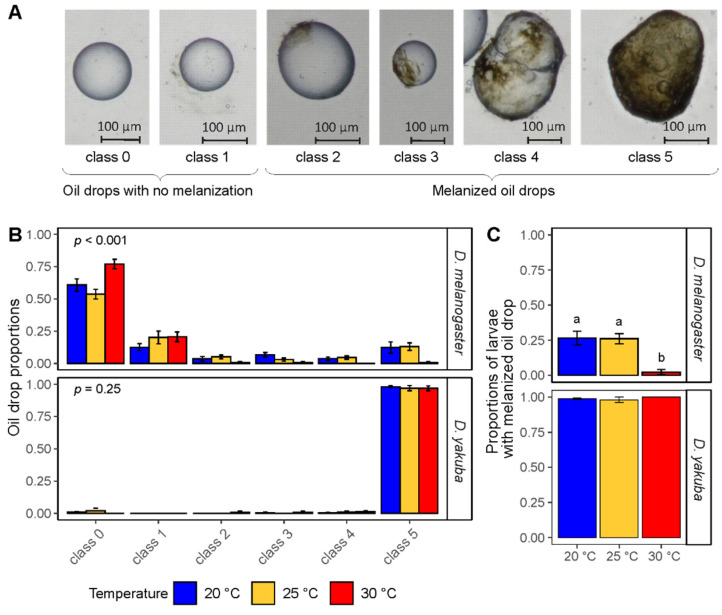
Impact of temperature on the host encapsulation capacity. (**A**) Example of oil drops with different degrees of encapsulation, ranging from class 0 = “no host response” to class 5 = “strong melanization response”. (**B**) Capacity of *D. melanogaster* and *D. yakuba* to encapsulate an oil drop as a function of temperature. Proportion of host larvae containing oil drops with different level of melanization. p on top left is the p-value from the Fisher Exact Test. (**C**) Capacity of *D. melanogaster* and *D. yakuba* to encapsulate an oil drop as a function of temperature. Proportion of host larvae able to induce a melanized response (i.e., containing oil drops with at least one melanization spot on the oil drop). Different letters indicate a significant difference between temperatures. Bars indicate standard errors (see Supplementary Tables S5 and S6 for sample sizes and statistical outputs).

Because the larval development and response times to oil injection differed between temperatures and the two *Drosophila* species, larvae maintained at 25 or 30 °C were dissected 48 and 72 h after oil injection for *D. yakuba* and *D. melanogaster* larvae, respectively. Those maintained at 20 °C were dissected one day later for both species. Among the *Drosophila* larvae containing an oil drop, six classes were defined to evaluate encapsulation capacity of the host: class 0 = “no host response”: no cell layer or melanization spot around the oil drop; class 1 = “host cellular response”: cell layer around the oil drop but no melanization spot; class 2 = melanization spots covering less than 25% of the oil drop; class 3 = melanization spots covering 25% to 50% of the oil drop; class 4 = melanization spots covering 50% to 75% of the oil drop; class 5 = melanization spots covering more than 75% of the oil drop. Classes 0 and 1 were considered as “oil drop with no melanization” whereas classes 2 to 5 were considered “melanized oil drop” (Figure 3A).

#### Statistical Analysis

The independence of the six classes defined above and the three temperatures was tested in R with the *fisher.test* function and the argument “simulate.p.value = TRUE”. Then, we fitted GLMMs with the number of larvae with a melanized oil drop versus the number of larvae without any melanization spot (corresponding to the host encapsulation capacity) as a response variable, temperature as a fixed factor, capillary as a random effect (since variation in the diameter of the capillary could significantly influence the melanic response of the host to the oil drop) and a binomial error distribution. As in Section 2.4., overdispersion was tested and GLMM followed by post-hoc Tukey tests were performed. See Supplementary Tables S5 and S6 for sample sizes and statistical outputs.

### 2.6. Analysis of the Parasitoid Venom Composition

The effect of temperature on the total amount of venom proteins and venom composition was assessed independently for *L. boulardi* ISm and ISy lines. For each line, five replicates were created with seven females per replicate and temperature (i.e., 105 females per line). Venom reservoirs were dissected individually in 15 µL of Ringer’s solution supplemented with a cocktail of protease inhibitors (PI; Roche Diagnostics GmbH, Mannheim Germany), mixed with an equivalent volume of Laemmli reducing buffer, and heated 10 min at 95 °C. The content of each reservoir was then divided in half, with one part used for global analysis and the other part for specific analysis. Protein separation was performed by 1D SDS-PAGE electrophoresis using commercial gels (8–16% Criterion™ TGX™ Precast Midi Protein Gel, Bio-Rad, Hercules, CA, USA).

Global analysis was performed according to the method described in [36] and used in [37,38]. Briefly, the gels were silver-stained [62] and photographed three times during the protein revelation step (digital camera EOS-5D-MkII, Canon, Tokyo, Japan). The resulting high-resolution pictures were analyzed with Phoretix-1D software (now CLIQS, TotalLab, Newcastle upon Tyne, UK) to extract the intensity profile of each lane. R functions were then used to obtain, for each lane, the intensities of a set of “reference bands” of known molecular weight for each parasitoid line. The intensity of these “reference bands” was normalized and estimated with the following combination of parameters: no background—peak volume—quantile normalization for *L. boulardi* ISm; no background—peak volume—cyclic loess normalization for *L. boulardi* ISy.

The specific analysis was performed using Western blots as described in Section 2.3.

#### 2.6.1. Statistical Analysis for the Global Analysis of Venom Composition

We first fitted linear mixed models (LMM) with the total band intensity in silver-stained gels (used as a proxy for the total amount of proteins in the venom) as a response variable, temperature as a fixed factor and replicate as a random effect. LMMs were fitted with the *lmer* function in the “lme4” R package [59] and followed by post-hoc Tukey tests as in Section 2.4 and Section 2.5.

To determine if venom protein composition was affected by temperature, we fitted a PERMANOVA for each parasitoid line (“vegan” R package; function *adonis2*) [63,64]. PERMANOVAs were fitted with all protein bands as response variables, temperature and replicates as fixed factors and 5000 permutations. To determine whether groups of individuals at 20 °C, 25 °C or 30 °C differed in venom composition for each parasitoid line, linear discriminant analyses (LDA) were performed with the “ade4” R package [65], using individual venom compositions as continuous variables and temperature as a factor. Since LDA does not account for variation between replicates, they were centered before the analysis using the *wca* function in the “ade4” R package [65]. With this additional step, all replicates had the same mean for each variable and, thus, the observed variation cannot result from variation between replicates. To describe the change in venom with temperature, an arrow representing the linear regression calculated from coordination of the three centroid points at 20, 25 and 30 °C was plotted for each parasitoid line.

Non-parametric Spearman rank correlation tests were performed to identify protein bands that correlated with the linear regression describing the trend of venom modification. P-values were Bonferroni corrected using the *p.adjust* R function. Because some protein bands were likely indirectly correlated with the linear regression due to their correlation with other protein bands, a combination of clustering and partial correlation analyses was used to identify bands for which their intensity changed directly with temperature. We first performed an UPGMA clustering analysis (“pvclust” R package; function *hclust*) with 1 − |ρ| as the metric distance, where ρ is the spearman correlation. Next, we used a correlation threshold of 0.45 to construct band clusters for which false detection of some bands as “correlated” could have occurred. For each cluster with at least two protein bands correlated to the regression, we performed partial correlations (*pcor* function in the “ppcor” R package) to determine if the residual variation in the intensity of each band, independent from the other bands in the cluster, was still correlated with the regression describing the venom change. *p*-values were also Bonferroni corrected using the *p.adjust* R function.

#### 2.6.2. Statistical Analysis for the Specific Analysis of Venom Composition

Because the amount of protein deposited can vary between gels and lanes, the relative intensities of LbSPNm, LbSPNy, LbGAP and LbGAP2 were calculated as the ratio of the signal intensity in Western blots for each of these proteins to the “total band intensity” in silver-stained gels. Linear mixed models (LMM) were fitted with either the relative intensity of LbSPNm in Ism, LbSPNy in ISy, LbGAP in ISm or LbGAP2 in ISm as a response variable, the temperature as a fixed factor and replicates as a random factor. For ISy, a Boxcox transformation was applied to limit the deviation of residuals from normality. LMM were fitted as described above and followed by Tukey tests. See Supplementary Table S7 for statistical outputs.

#### 2.6.3. Identification of Venom Proteins

The attempt to identify the proteins contained in the temperature-affected bands was achieved by matching these bands with those of the 1D electrophoresis gels used for the previous proteomics of *L. boulardi* ISm and ISy venom [29]. The intensity level of a band may result from that of several proteins having migrated to the same position. However, the proteins responsible for the high intensity of a band are likely to be the most abundant proteins in that band. Therefore, we used the peptide match number from the previous mass spectrometry study [29] to classify the proteins in the bands as abundant or not.

## 3. Results

### 3.1. Impact of Temperature during Parasitism on the Outcome of the Interaction

We first tested the effect of exposing the two partners of the *Drosophila-L. boulardi* interaction to different temperatures during the first 48 to 72 h after oviposition. Parasitism experiments involved *D. yakuba* and *D. melanogaster* hosts, and the ISm and ISy lines of *L. boulardi*, at 20 °C, 25 °C and 30 °C.

We tested the effect of temperature on: (i) the parasitism rate (mono- and multi-parasitized host larvae among all host larvae); (ii) the parasitic success (i.e., the proportion of mono-parasitized host larvae containing a free parasitoid larva alone or with an open capsule); (iii) the capacity of the host to encapsulate the parasitoid egg (i.e., the proportion of mono-parasitized host larvae containing a complete or open capsule); (iv) the parasitoid capacity to escape from a capsule (i.e., the proportion of host larvae containing a free parasitoid larva together with an open capsule among mono-parasitized hosts showing an encapsulation response) (Figure 2A).

No effect of temperature was observed on the parasitism rate (Figure 2B, Tukey tests, *p* > 0.30 for all), suggesting an absence of effect of the temperature at which parasitism occurs on the ability of *L. boulardi* to parasitize host larvae. Four host–parasitoid interactions were analyzed in the experiment. No effect of temperature on parasitic success—always close to 100%—and host encapsulation capacity—close to 0%—was observed for ISm—*D. melanogaster* and ISy—*D. yakuba* interactions (Figure 2C). In contrast, the parasitic success increased with temperature for both ISm—*D. yakuba* and ISy—*D. melanogaster* interactions although no significant difference was observed between 25 and 30 °C for ISy—*D. melanogaster* interaction (Figure 2C, Tukey tests, *p* = 0.07 between 25 and 30 °C for ISy—*D. melanogaster*, *p* < 0.01 for others). The success of ISm seems to result solely from its capacity to escape from a capsule since the capacity of *D. yakuba* to encapsulate ISm remains at 100% regardless of temperature (Figure 2C, Tukey tests, *p* < 0.01). In contrast, the success of ISy appears to result from both a decrease in *D. melanogaster* encapsulation capacity with higher temperature (Figure 2C, Tukey tests, *p* < 0.001) and a greater ability of ISy to escape from a capsule at least at 25 °C (Figure 2C, Tukey tests, *p* < 0.01).

### 3.2. Temperature Effect on the Host Capacity to Encapsulate

The effect of temperature on the host encapsulation capacity alone was assessed by injecting paraffin oil in *D. yakuba* or *D. melanogaster* larvae prior to their exposure to 20, 25 or 30 °C. Following dissection, six classes of encapsulation, ranging from class 0 = “no host response” to class 5 = “melanization spots covering more than 75% of the oil drop” were recorded (Figure 3A). When multiple oil drops were found in a *Drosophila* larva, only the one belonging to the highest encapsulation class was considered.

The proportions of each melanization class varied with temperature only for *D. melanogaster* (Figure 3B, FET, *p* < 0.001 for *D. melanogaster; p* = 0.55 for *D. yakuba*). We then tested the effect of temperature on the ability of each host to induce a melanized response (class pool 2 to 5). We confirmed the ability of *D. yakuba* to induce a melanization response most of the time, regardless of temperature (Figure 3C, X^2^ = 2.8, *p* = 0.25). The melanization ability of *D. melanogaster* was reduced at 30 °C compared to 20 and 25 °C (Figure 3C, Tukey tests, *p* < 0.001).

### 3.3. Impact of Temperature during Parasitoid Nymphal Development on Interaction Outcome

In a second experiment, we tested whether temperature during parasitoid nymph development affects (i) the parasitism rate, (ii) the parasitic success, (iii) the capacity of the parasitoid to inhibit capsule formation (i.e., the proportion of mono-parasitized host larvae containing a free parasitoid larva alone) and (iv) the capacity of the parasitoid to escape from a capsule (Figure 2A). As a preliminary step, we identified that venom protein synthesis begins after 18 and 20 days of development in ISm and ISy, respectively (Supplementary Figure S1). Parasitoid nymphs were therefore exposed to 20 °C, 25 °C or 30 °C during a period ranging from one day before venom protein detection to two days after adult emergence (Figure 1). Parasitic tests were then performed at 25 °C with *D. yakuba* and *D. melanogaster* host species.

**Figure 4:**
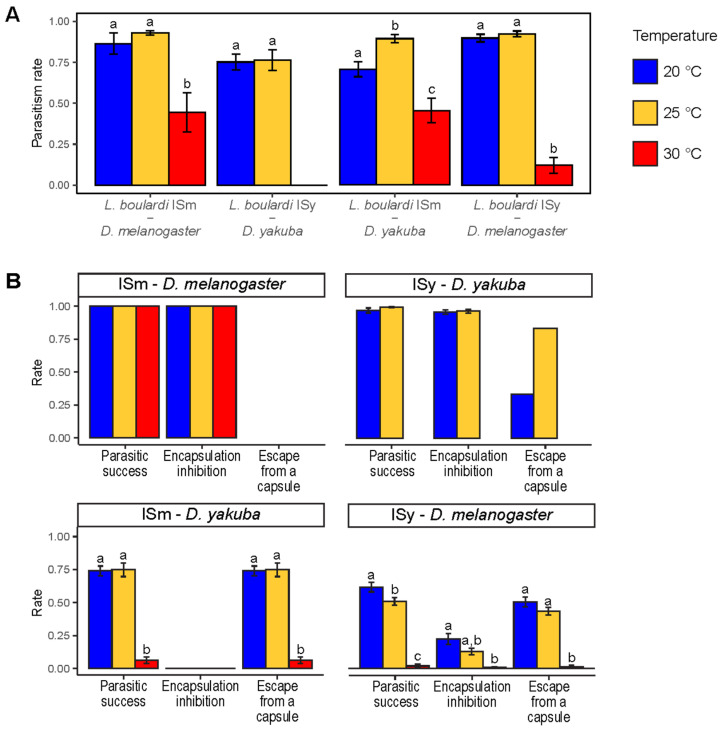
Impact of temperature during parasitoid nymphal development on the outcome of host–parasitoid interaction. (**A**) Parasitism rates for the four host–parasitoid interactions as a function of temperature. (**B**) Rate of (i) parasitic success, (ii) parasitoid capacity to inhibit encapsulation by the host and (iii) parasitoid capacity to escape from a capsule, depending on the temperature. Within each host—parasitoid interaction, different letters indicate a significant difference between temperatures for a given parameter. Bars indicate standard errors. There are no error bars for the parasitoid capacity to escape from a capsule for the ISy—*D. yakuba* interaction at 20 °C et 25 °C because only one and two host larvae contained a capsule, respectively, and it was an open capsule (see Supplementary Tables S3 and S4 for sample sizes and statistical outputs).

For all four *L. boulardi*—host interaction, the parasitism rate was lower when parasitoid nymphs were developed at 30 °C than at the other two temperatures (Figure 4A, Tukey tests, *p* < 0.001). This negative effect of temperature on parasitism rate was stronger for *L. boulardi* ISy than ISm, with very few larvae parasitized by ISy females reared at 30 °C for *D. melanogaster* (from 18 parasitic tests within total 524 larvae, we found at least one parasitized larva in only nine parasitic tests for a total of 61 parasitized larvae) and none for *D. yakuba* (Figure 4A). In contrast, no difference in parasitism rate was observed between parasitoids reared at 20 °C or 25 °C, except for the interaction between ISm and *D. yakuba* for which the parasitism rate was higher for parasitoids reared at 25 °C (Figure 4A, Tukey tests, *p* = 0.022).

The parasitic success of ISm on *D. melanogaster* and ISy on *D. yakuba*—resulting primarily from the ability of the parasitoid to inhibit host encapsulation—remained close to 100% regardless of temperature (Figure 4B, no variation for ISm—*D. melanogaster*, 20 °C vs. 25 °C, *p* = 0.055 for ISy—*D. yakuba*).

In contrast, parasitic success was strongly reduced when parasitoids were reared at 30 °C compared to 20 °C or 25 °C for ISm—*D. yakuba* and ISy—*D. melanogaster* interactions (Figure 4B, Tukey tests, *p* < 0.001). In the latter interaction, a slight decrease in parasitic success was also observed between 20 °C and 25 °C (Figure 4B, Tukey test, *p* = 0.006). For the ISm—*D. yakuba* interaction, the decrease in parasitic success appeared to result from a decrease in the ability of ISm to escape from a capsule, as no inhibition of encapsulation capacity was observed (Figure 4B). For the ISy—*D. melanogaster* interaction, the decrease in parasitic success appeared to result from a decrease in both the ability of ISy to escape from a capsule and to inhibit encapsulation (Figure 4B, Tukey tests, 20 °C vs. 30 °C, *p* = 0.02 for capsule inhibition, 20 °C vs. 30 °C and 25 °C vs. 30 °C, *p* < 0.001 for escape capacity).

### 3.4. Impact of Temperature during Parasitoid Nymphal Development on Venom Composition

The effect of temperature on ISm and ISy venom was evaluated on the amount and protein composition of 35 females per temperature and line. A total of 210 females were analyzed by the global approach with 35 and 32 reference bands identified for ISm and ISy, respectively, whose intensities represent the variables describing the protein composition of the venom.

**Figure 5:**
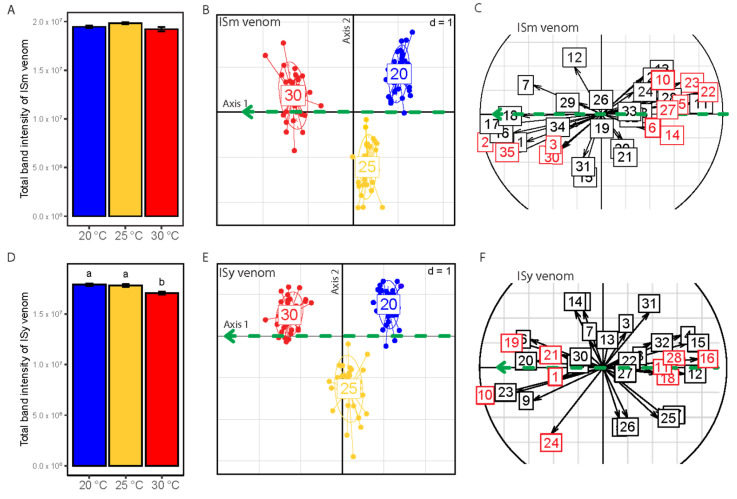
Impact of temperature on venom during parasitoid nymphal development. (**A**,**D**) Total intensity of venom bands (proxy of the amount of protein in venom) in ISm (**A**) or ISy (**D**). Different letters indicate a significant difference between temperatures. Bars indicate standard errors. (**B**,**E**) Position of *L. boulardi* ISm (**B**) or ISy (**E**) individuals on the two discriminant axes. Individuals are grouped and colored according to the rearing temperature (20 °C, 25 °C and 30 °C). The green arrows represent the trend of the venom change with increasing temperature (i.e., linear regressions calculated from the coordinates of the three centroid points corresponding to the three temperatures). (**C**,**F**) Correlation circles indicating the correlation of protein bands with linear regressions in ISm (**C**) or ISy (**F**). The green arrows are the same as in (**B**,**E**). The numbers correspond to the protein bands. Bands in red are significantly correlated with linear regressions, meaning that their intensity varies with temperature. See Supplementary Tables S8–S11 for statistical outputs.

We first tested the impact of temperature on the amount of protein in the venom for each temperature and parasitoid line using the total band intensity (sum of intensities of all identified the reference bands) as a proxy. No difference was observed in ISm parasitoids (Figure 5A, F_2,12_ = 1.9, *p* = 0.19), while ISy parasitoids reared at 30 °C showed lower total band intensity than those reared at 20 or 25 °C (Figure 5D, *p* < 0.01 for both pairwise comparisons involving 30 °C).

Considering the protein composition of the venom, temperature has a significant overall effect on the protein band intensity for both parasitoid lines (Supplementary Table S9, *p* < 0.001 for both lines). Developmental temperature explained 35% and 32% of the variation in venom composition in ISm and ISy lines, respectively, whereas replicates (corresponding to different starting vials), although significant, accounted for only 6% of the variation in venom composition (Supplementary Table S9). Replicates were centered to remove their effect before further characterizing the impact of temperature on venom protein composition. Linear discriminant analysis (LDA) performed on the residual variation in band intensity, separating individuals reared at 20 °C, 25 °C or 30 °C into three distinct non-overlapping groups (Figure 5B,E). Both PERMANOVA and LDA results therefore suggest that temperature affects not only the total amount of protein in ISy, but also the protein composition of the venom of both parasitoid lines.

### 3.5. Identification of Venom Proteins Impacted by Temperature

Protein bands for which the intensity changed with temperature were identified on the basis of their correlation to the linear regressions calculated from the centroid points of the temperature groups and represented by green arrows. Some of these protein bands could have been misidentified as influenced by temperature because of their correlation with other bands, either because of their proximity of migration on the gel or to a linkage disequilibrium. A combination of clustering (Supplementary Figure S2) and partial correlation analyses was therefore applied to identify protein bands that were directly impacted by temperature.

The analysis revealed that the intensity of 11 *L. boulardi* ISm venom protein bands changed with temperature. Of these 11 bands, the intensity of four increased at higher temperature and the intensity of seven at lower temperature (Figure 5C). Concerning ISy venom, the intensity of nine proteins bands changed with temperature, with five for which the intensity increased at higher temperature and four for which the intensity decreased at higher temperature (Figure 5F).

The temperature-affected protein bands were matched with those of the 1D electrophoresis gels used for the previous proteomics of *L. boulardi* ISm and ISy venom [29]. This allowed for identification of at least one abundant protein for six out of the 11 temperature-affected bands for *L. boulardi* ISm and seven out of the nine temperature-affected bands for *L. boulardi* ISy (Table 1 and Table 2). Although their coding sequence was previously determined [29], the most abundant proteins in a majority of these bands had no similarity to known proteins (Table 1 and Table 2). Their biochemical function therefore remains to be determined, as is true for a majority of the venom proteins of *L. boulardi* ISm and ISy [29].

**Figure 6:**
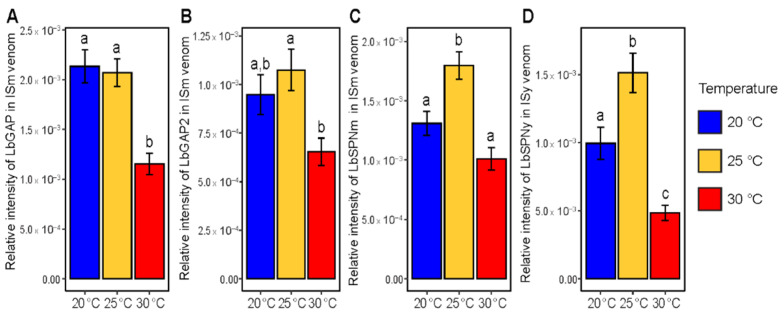
Impact of temperature during parasitoid nymphal development on the amount of specific proteins in the venom. Relative amount of LbGAP (**A**), LbGAP2 (**B**) and LbSPNm (**C**) in *L. boulardi* ISm venom and of LbSPNy in *L. boulardi* ISy venom (**D**). Different letters indicate a significant difference between temperatures. Bars indicate standard errors.

For *L. boulardi* ISm, LbGAP was identified as the most abundant protein in band #22 and LbGAP2 as the most abundant protein in band #27, with the amount of both decreasing with increasing temperature (Table 1). The decrease in the amount of LbGAP with temperature was confirmed by the specific analysis with antibodies (Figure 5, *p* < 0.01 for pairwise comparisons involving 30 °C). The specific analysis also showed a reduction in the amount of LbGAP2 for parasitoids reared at 30 °C, although it was only significant between 25 °C and 30 °C (Figure 6, *p* = 0.017) and marginally significant between 20 and 30 °C (Figure 6, *p* = 0.078). Finally, the specific analysis showed an increase in the amount of LbSPNm at 25 °C, whereas the global analysis failed to detect this change. This difference could be due to the fact that, in the global analysis, we examined protein bands correlated to the linear regression, i.e., protein bands whose intensity changed linearly with temperature. Therefore, protein bands with different intensity at 25 °C only could not be detected by the global approach.

For *L. boulardi* ISy, we could identify LbSPNy as the most abundant protein in band #18 and/or 19 for which the intensity changed differently with temperature (Figure 5F, Table 2). The specific analysis showed a decrease in LbSPNy at 30 °C and an increase at 25 °C (Figure 5, *p* = 0.009 for the comparison between 20 and 30 °C, *p* < 0.001 for the comparison between 25 and 30 °C and *p* = 0.033 for the comparison between 20 and 25 °C). As with LbSPNm in ISm, the amount of LbSPNy did not change linearly with temperature, which could explain the opposite results found by the global approach for bands #18 and 19. LbGAPy4 was found to be the most abundant protein in band #24 whose intensity increased with temperature (Table 2).

## 4. Discussion

Although many studies have shown parasitoid disadvantage at elevated temperatures [16,17,18,19,20,21,22,66,67], parasitoid success increased with temperature for *L. boulardi* ISm—*D. yakuba* and *L. boulardi* ISy—*D. melanogaster* interactions. A similar trend was observed for another *D. melanogaster* line selected for increased resistance to a Turkish population of *L. boulardi* using temperatures ranging from 15 °C to 25 °C [17]. The increase in parasitic success at a higher temperature seems to be specific to *L. boulardi*—*D. melanogaster* and *D. yakuba* interactions. Indeed, the opposite result occurred for *L. boulardi*—*D. simulans* [18] and for *L. heterotoma* and *Asobara tabida* on different *Drosophila* host species [16,17,18,21,67].

Our results suggest that the mechanisms underlying the increase in parasitic success with temperature differ between ISm—*D. yakuba* and ISy—*D. melanogaster* interactions. In the first interaction, the increase in parasitic success results solely from the increased ability of the parasitoid to escape from the capsule, whereas, in the second case, it is due to this same effect at 25 °C combined with the decreased encapsulation capacity of the host with increasing temperature. The increased escape ability could be due to an increased amount of proteins in the venom injected by *L. boulardi* into host larvae, either because of a higher expression of encoding genes and/or their translation with temperature, to an increased efficiency of venom proteins with increasing temperature or temperature-dependent post-translational modifications. The involvement of secretions from the parasitoid larva itself is rather unlikely, as this has never been reported for *L. boulardi*, unlike other parasitoids [68,69]. Finally, the increase in parasitoid escape capacity may also result from a negative effect of temperature on the production of toxic radicals such as ROS and RNS that are generated by the host phenoloxidase cascade during the encapsulation process and are presumed to be responsible for the death of the parasitoid larva [32].

The injection of paraffin oil allowed us to evaluate the immune response of both *Drosophila* species independently of the parasitoid and depending on temperature. Almost all oil-injected *D. yakuba* larvae contained fully encapsulated drops, regardless of temperature, suggesting no impact on the immune response of this species. Contrariwise, the majority of *D. melanogaster* larvae did not respond or responded only weakly to oil drops. Similarly, a higher rate of encapsulation of *Asobara tabida* eggs was reported for *D. yakuba* compared to *D. melanogaster* [70]. The reduced ability of *D. melanogaster* to encapsulate a foreign body at 30 °C was therefore observed independently of parasitism. However, although a decrease in encapsulation capacity was observed between 20 and 25 °C in response to ISy eggs, no change occurred for encapsulation of oil drops. All this suggests that temperature impacts both interacting partners, although a direct host effect on parasitoid success cannot be excluded, via a negative temperature effect on capsule formation and toxic radical production. The difference in temperature impact between the two *Drosophila* species may result from a higher basal encapsulation capacity of *D. yakuba*, but also from a higher level of induction of immunity in response to oil injection.

Various studies have examined the influence of temperature on the immunity of *D. melanogaster*. For example, different genotypes of *D. melanogaster* sampled in Africa and North America were tested for resistance to bacterial infection and fecundity at three different temperatures [71]. While all populations showed a lower bacterial load and higher fecundity at 28 °C than at cooler temperatures, variations between genotypes were observed. African flies had lower immunity and fecundity than North American flies at the lowest temperature [71]. Therefore, immune variations that we observed between *D. melanogaster* and *D. yakuba* could also have happened at the intra-specific level. Another study tested the relation between temperature, cuticle color (melanin amount) and immunity against bacteria [72]. The authors showed that flies reared in a warmer environment harbored lighter cuticles and lower resistance to pathogens compared to flies reared in a cooler environment. Finally, Ref. [73] examined the effects of temperature on *D. melanogaster* resistance against two bacterial species. The authors observed lower host resistance at a higher temperature, probably due to enhanced bacterial growth at high temperatures and altered expression of immune-related genes in the host. Altogether, this suggests the occurrence of temperature-specific effects on the host and/or its pathogen. Our data here are consistent with this conclusion; however, this time considering the response to an endoparasitoid.

Part of our work investigated the impact of parasitoid rearing temperature on venom composition while testing whether such a temperature-induced change could be related to a change in parasitic success. Developmental temperature had a significant impact on venom composition of both ISm and ISy parasitoid lines. More than 30% of the variation in venom composition was indeed explained by developmental temperature. For comparison, changes in *L. boulardi* venom composition in response to host selection accounted for less than 10% of the variation in our previous studies [37,38]. Of the ISm proteins whose quantity in the venom was impacted by temperature, only LbGAP has a defined function. LbGAP is thought to be required for the parasitic success of ISm on *D. melanogaster* by inducing morphological changes in host immune cells dedicated to encapsulation [42,43,44,45,46]. Although the amount of LbGAP in venom was reduced approximately by half at 30 °C, no change was observed in the parasitic success of ISm on *D. melanogaster.* However, a previous experiment showed that a 50% reduction in the amount of LbGAP in the venom of F_1_ ISm/ISy hybrids had no impact on parasitic success on *D. melanogaster* [42].

The parasitic success of ISm on *D. yakuba* decreased at 30 °C. It is unlikely that this reduction is due to the decrease in the amount of LbGAP in the venom. Indeed, LbGAP would act to suppress encapsulation, whereas our results suggest a mechanism based on capsule escape for ISm in *D. yakuba*. The amount of LbGAP2 in ISm venom was also found to be reduced at 30 °C. LbGAP2 may play a role in the parasitic success of ISm; however, this remains to be demonstrated even though one of our previous study suggests a selection of this protein on *D. yakuba* [38]. LbGAP2 is also a RhoGAP; however, the presence of a mutation in the active site makes its function difficult to predict. Proteins of unknown function found in other decreasing bands could also be responsible for the decrease in parasitic success of ISm on *D. yakuba*. However, caution must be exercised regarding this observed decrease in parasitism success at 30 °C, since rearing *L. boulardi* females at this temperature appears to negatively affect their overall physiology. Few adult individuals emerged, and their parasitism rate was greatly reduced and even fell to zero for the ISy—*D. yakuba* interaction. High temperatures can be costly for insects [5] and, due to stress, organisms may change their resource allocation [74]. Several studies on other parasitoids have shown reduced fecundity under extreme temperatures during development [75,76,77,78]. We can therefore hypothesize that *L. boulardi* females have reallocated their energy towards survival instead of reproductive traits. Another explanation for the reduced rate of parasitism would be a decrease in the amount of venom. However, the amount of venom was not affected by temperature for ISm and only slightly decreased for ISy, making this hypothesis unlikely. Therefore, we cannot rule out that the decrease in success at 30 °C was more related to a change in the overall physiology of ISm females than in the venom composition.

No change in parasitic success occurred for ISy on *D. yakuba* as a function of the parasitoid rearing temperature, whereas ISy parasitic success on *D. melanogaster* decreased between each temperature tested. Among the ISy proteins whose quantity in the venom was impacted by temperature, only LbSPNy has a predicted function. LbSPNy is known to inhibit the activation of the phenoloxidase cascade—a crucial step for the melanization process—in *D. yakuba* [47]. Nothing is known about its effect on *D. melanogaster*; however, a previous study showed that LbSPNy was counter-selected on *D. melanogaster* [38]. However, the amount of LbSPNy in ISy venom increased between 20 °C and 25 °C, and decreased between 25 °C and 30 °C. Therefore, it is unlikely that the change in the amount of LbSPNy in the venom explains the decrease in parasitic success of ISy on *D. melanogaster*. It is therefore possible that this decrease is due to one or more proteins whose amount in the venom is impacted by temperature, but whose function is unknown.

Surprisingly, LbSPNy was found in two different protein bands (#18 and 19) with opposite trends. This can be explained by the fact that the global approach was able to identify protein bands whose intensity changes linearly with the temperature while the specific analysis showed an increase in the amount of LbSPNy at 25 °C, which is the intermediate temperature. It is possible that band #18 mainly reflects the change between 25 °C and 30 °C, while band #19 reflects the change between 20 °C and 25 °C.

A venom change due to temperature may also affect other organisms than parasitoids that depend on venom production for survival, especially for killing prey or enemies. Temperature effects could affect the amount of venom produced, the composition of this venom or even its level of toxicity [79,80,81]. As with the evolution of venom, comparisons between venomous organisms could help in identifying common mechanisms to venomous organisms linking temperature to venom changes.

Our study highlights a probable effect of the temperature on the *Drosophila—L. boulardi* immune interaction but also independently on the two partners. To further investigate the impact of temperature on *Drosophila* immune defenses, we could evaluate its effect on the cellular and humoral components of encapsulation. For example, we could compare the impact of temperature before and after parasitism on specific components of the immune response, such as the number of hemocytes, phenoloxidase activity or the expression of genes encoding PPO2 and PPO3, two enzymes involved in melanization [82].

## 5. Conclusions

Our results suggest that general predictions on the impact of temperature on parasitoid–host interactions are difficult and must be made for each species–species interaction. Indeed, although increasing temperature most often favors the host, the opposite has been observed in this study. A change in parasitic success was observed for ISy which could be related to the demonstrated impact of temperature during parasitoid development on the venom composition. The relationship between rearing temperature, venom composition and parasitic success is particularly important in the context of biological control. Indeed, a change in the temperature at which parasitoids are reared before being released in the field as auxiliaries could directly impact the success of biological control. Although it is easy to find 30 °C in the natural environment, we are aware that it is rare to have it constantly. It would therefore be interesting to repeat our experiments under fluctuating temperatures, since temperature fluctuates daily in a natural environment and responses could be quite different in the two conditions [19,83,84]. In addition, a change in temperature may extend over several generations. Instead of focusing on the plasticity of the response, one could test (i) whether the change in venom composition under different rearing temperatures persists in offspring reared at the same temperature, as has been done for some other traits and species [85], and (ii) whether venom composition and parasitic success can evolve under selection in response to different temperatures. Finally, we showed that venom composition was more variable in response to rearing temperature than to selection by the host. This raises the question of whether the study of parasitoid venom composition in the field reflects a local adaptation [39], a plastic response or both.

## Figures and Tables

**Table 1:** Match between temperature-affected ISm protein bands and their putative protein content determined from comparison with data from [29]. Only proteins for which at least 10 peptide matches were found in Mascot searches with unisequences identified in the venom apparatus transcriptomics [29] were considered abundant and were therefore listed. The number of proteins found in the band, their predicted function and the number of peptide matches for each unisequence are provided. Up (“↗”) and down (“↘”) arrows indicate an increase or decrease in band intensity in response to increasing temperature. NA: data not available.

Reference Band	Number of Abundant Proteins	Putative Function	Number of Peptide Matches	Band Intensity in Response to Increasing Temperature
2	1	Unknown	12	↗
3	2	Unknown	33	↗
		Unknown	34	
5	0	NA	NA	↘
6	0	NA	NA	↘
10	0	NA	NA	↘
14	2	Unknown	39	↘
		Unknown	36	
22	5	RhoGAP (LbGAP)	52	↘
		Unknown	21	
		Serpin (LbSPNm)	17	
		RhoGAP	11	
		(LbGAPy)		
23	NA	NA	NA	↘
(not analyzed in [29])				
27	2	RhoGAP (LbGAP2)	20	↘
		Unknown	18	
30	1	Unknown	19	↗
35	0	NA	NA	↗

**Table 2:** Match between temperature-affected ISy protein bands and their putative protein content determined from comparison with data from [29]. Only proteins for which at least 10 peptide matches were found in Mascot searches with unisequences identified in the venom apparatus transcriptomics [29] were considered abundant and were therefore listed. The number of proteins found in the band, their predicted function and the number of peptide matches for each unisequence are provided. Up (“↗”) and down (“↘”) arrows indicate an increase or decrease in band intensity in response to increasing temperature. NA: data not available.

Reference Band	Number of Abundant Proteins	Putative Function	Number of Peptide Matches	Band Intensity in Response to Increasing Temperature
1	NA	NA	NA	↗
(not analyzed in [29]				
10	2	Unknown	10	↗
		Unknown	10	
11	0	NA	NA	↘
16	1	Unknown	16	↘
18 and 19	1	Serpin (LbSPNy)	81	↘(18)
(not separated in [29])				↗(19)
21	1	Unknown	25	↗
24	1	RhoGAP (LbGAPy4)	24	↗
28	2	Unknown	20	↘
		RhoGAP (LbGAPy2)	17	

## Data Availability

The datasets supporting the results are available in the FigShare repository under the doi:10.6084/m9.figshare.14706465.

## Supplementary Materials

The following are available online at https://www.mdpi.com/article/10.3390/insects12070647/s1, Figure S1: Assessment of the developmental stage of *L. boulardi* at which synthesis of venom proteins begins, Figure S2: Clustering analysis of protein bands of ISm (left) and ISy (right) parasitoids, Table S1: Sample size for the “experiment 1” (effect on interaction), Table S2: Summary of pairwise contrasts (outcome of the host-parasitoid interaction), Table S3: Sample size for the “experiment 2” (effect on parasitoid), Table S4: Summary of pairwise contrasts (outcome of the host-parasitoid interaction), Table S5: Sample size for the host capacity to encapsulate an oil drop (effect on host), Table S6: Summary of pairwise contrasts (melanized response to the oil drop), Table S7: Summary of pairwise contrasts (relative intensity of LbGAP, LbGAP2 and LbSPN). Table S8: Summary of pairwise contrasts (total band intensity), Table S9: PERMANOVA for venom variation in each parasitoid line, Table S10: Correlation values of *L. boulardi* ISm bands to linear regression (arrow from LDA) before and after partial correlation analysis, Table S11: Correlation values of *L. boulardi* ISy bands to linear regression (arrow from LDA) before and after partial correlation analysis.
