# Supplementary material for: Impact of Temperature on the Immune Interaction between a Parasitoid Wasp and Drosophila Host Species
Source: Insects. 2021 Jul 15;12(7):647. doi: 10.3390/insects12070647 (PMC8303993; doi:10.3390/insects12070647)
Supplement: Supplementary file 1 [file insects-12-00647-s001.zip › insects-1264594-supplementary.pdf]

Impact of temperature on the immune interaction between a parasitoid wasp and *Drosophila* host species

Fanny Cavigliasso[1,ǂ], Jean-Luc Gatti, Dominique Colinet[1,$] and Marylène Poirié [1,$,*]

SUPPLEMENTARY FIGURES

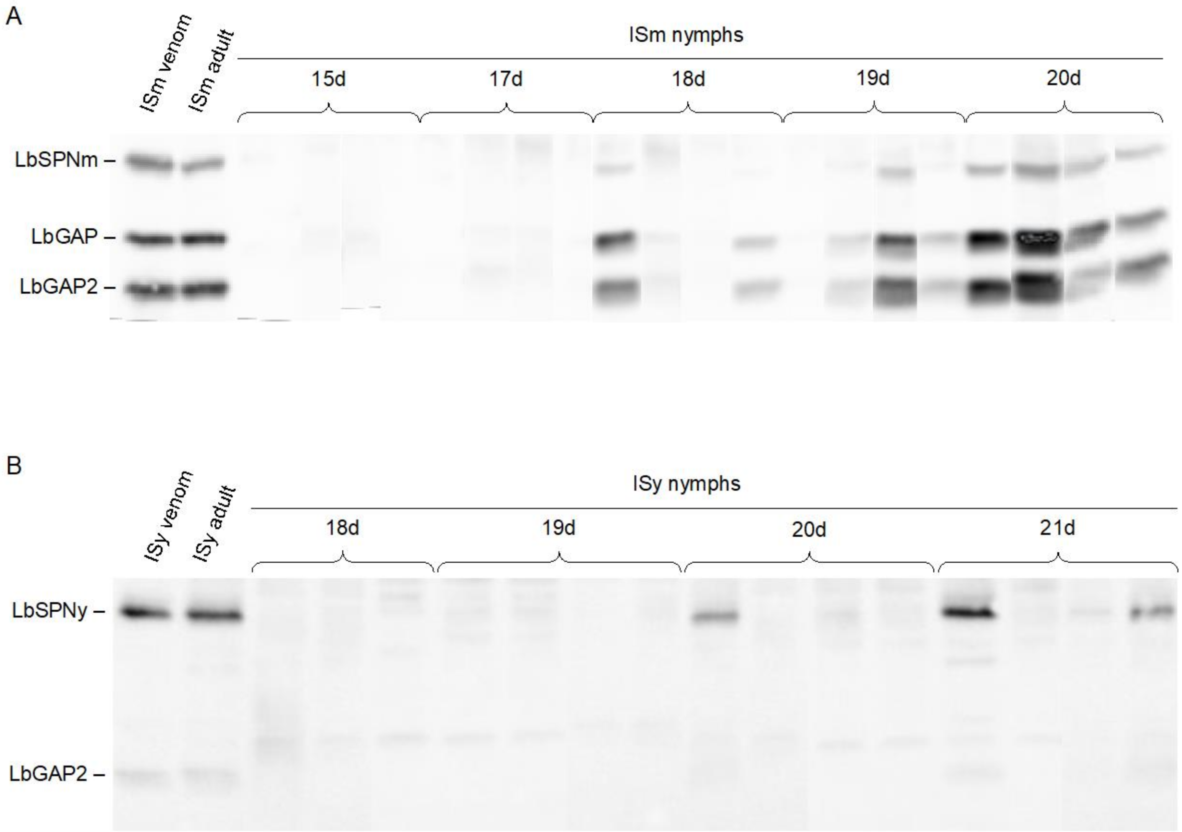

Supplementary Figure S1. Assessment of the developmental stage of *L. boulardi* at which venom proteins synthesis begins. Western blots were used for detection of venom proteins LbGAP, LbGAP2 and LbSPN with specific antibodies. (A) *L. boulardi* ISm line. (B) *L. boulardi* ISy line. Venom: lane containing venom from females of the corresponding line; Adult: lane containing the crushed abdomen of an adult female of the corresponding line; Nymph: lanes containing the crushed abdomen of 15 to 20-day-old or 18 to 21-day-old *L. boulardi* nymphs (ISm and ISy, respectively).

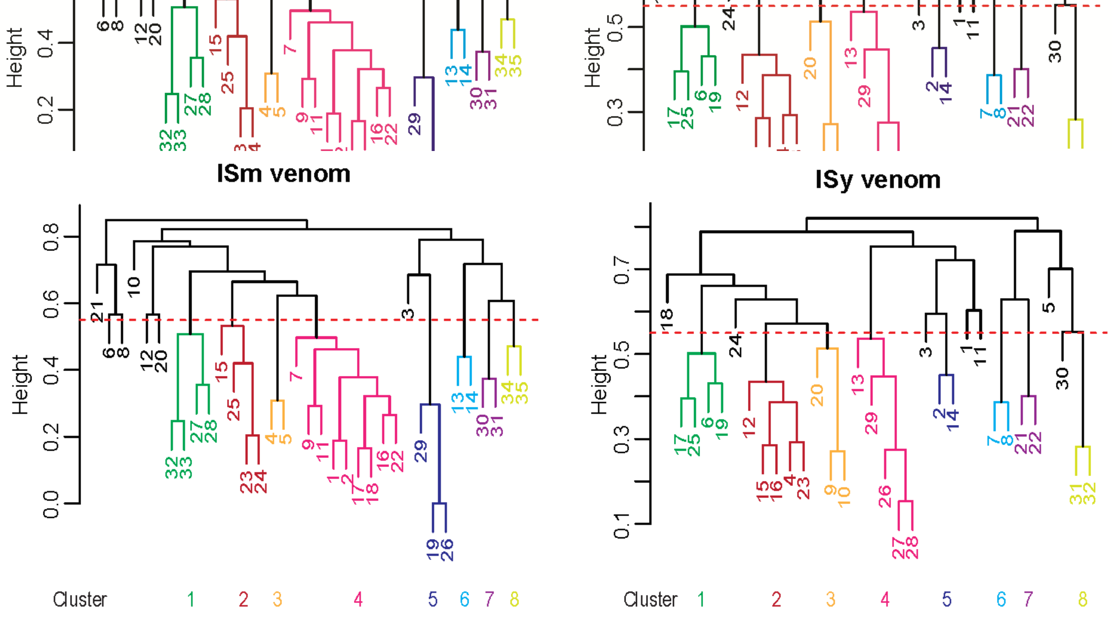

Supplementary Figure S2. Clustering analysis of ISm (left) and ISy (right) parasitoid protein bands. Each numbered leaf in the dendrogram corresponds to a venom protein band. The height represents the distance between bands calculated as “1 - (absolute value of correlation between bands)”. The horizontal lines at 0.55 therefore represent the correlation threshold of 0.45 used to build the clusters for the partial correlation analysis. The bands in black do not belong to any cluster. The bands colored with the same color belong to the same cluster.

Supplementary Tables

Supplementary Table S1. Sample size for the experiment on the impact of temperature during parasitism on the outcome of the interaction (see results section 3.1.). N represents the number of parasitic tests (i.e. the number of female parasitoids). The total number of larvae represents the number of larvae analyzed. For the parasitism rate, this is all larvae (alive and dissected); for the other parameters it is the number of mono-parasitized larvae only. The difference in the total number of larvae between the parasitism rate and the other parameters may be due to non-parasitized larvae but also to multi-parasitized larvae.

Host	Parasitoid	Parameter(s)	Temperature	N	Total number of larvae
D. melanogaster	ISm	Parasitism rate (Figure 2B)	20°C	5	132
			25°C	3	88
			30°C	5	150
		Parasitic success, host encapsulation capacity, escape from a capsule (Figure 2C)	20°C	5	81
			25°C	3	54
			30°C	5	65
D. melanogaster	ISy	Parasitism rate (Figure 2B)	20°C	13	372
			25°C	14	403
			30°C	14	410
		Parasitic success, host encapsulation capacity, escape from a capsule (Figure 2C)	20°C	13	295
			25°C	14	319
			30°C	14	303
D. yakuba	ISm	Parasitism rate (Figure 2B)	20°C	13	350
			25°C	13	339
			30°C	13	320
		Parasitic success, host encapsulation capacity, escape from a capsule (Figure 2C)	20°C	13	184
			25°C	13	200
			30°C	13	175
D. yakuba	ISy	Parasitism rate (Figure 2B)	20°C	6	169
			25°C	7	198
			30°C	8	227
		Parasitic success, host encapsulation capacity, escape from a capsule (Figure 2C)	20°C	6	103
			25°C	7	135
			30°C	8	142

Supplementary Table S2. Summary of pairwise contrasts comparing the effect of temperature during parasitism and the following 48 or 72 hours on the outcome of the host-parasitoid interaction, reported in Figure 2. When no variance was observed for a specific interaction and outcome, no statistical test was performed on it.

interaction	parameter	comparison	estimate	std. error	z ratio	p-value
ISm *D. yakuba*	Parasitism rate	20°C – 25°C	-0.35	0.27	-1.30	0.39
		20°C – 30°C	0.04	0.27	0.13	0.99
		25°C – 30°C	0.39	0.27	1.42	0.33
ISy *D. yakuba*	Parasitism rate	20°C – 25°C	-0.28	0.23	-1.22	0.44
		20°C – 30°C	0.01	0.22	0.04	1.00
		25°C – 30°C	0.29	0.21	1.36	0.36
ISm *D. melanogaster*	Parasitism rate	20°C – 25°C	1.13	0.85	1.33	0.38
		20°C – 30°C	0.67	0.72	0.94	0.62
		25°C – 30°C	-0.46	0.83	-0.55	0.85
ISy *D. melanogaster*	Parasitism rate	20°C – 25°C	0.05	0.35	0.13	0.99
		20°C – 30°C	0.39	0.35	1.12	0.50
		25°C – 30°C	0.34	0.34	1.00	0.58
ISm *D. yakuba*	Parasitic success	20°C – 25°C	-1.26	0.32	-4.02	<0.001
		20°C – 30°C	-2.03	0.31	-6.57	<0.001
		25°C – 30°C	-0.77	0.25	-3.08	0.006
ISm *D. yakuba*	Parasitoid escape capacity	20°C – 25°C	-1.26	0.32	-4.02	<0.001
		20°C – 30°C	-2.03	0.31	-6.57	<0.001
		25°C – 30°C	-0.77	0.25	-3.08	0.006
ISy *D. melanogaster*	Parasitic success	20°C – 25°C	-1.38	0.20	-6.93	<0.001
		20°C – 30°C	-1.74	0.20	-8.68	<0.001
		25°C – 30°C	-0.36	0.16	-2.19	0.072
ISy *D. melanogaster*	Host encapsulation capacity	20°C – 25°C	-1.36	0.35	-3.91	<0.001
		20°C – 30°C	-2.76	0.33	-8.38	<0.001
		25°C – 30°C	-1.39	0.20	-6.85	<0.001
ISy *D. melanogaster*	Parasitoid escape capacity	20°C – 25°C	-1.29	0.23	-5.67	<0.001
		20°C – 30°C	-0.60	0.26	-2.27	0.060
		25°C – 30°C	0.69	0.23	3.03	0.007

Supplementary Table S3. Sample size for the experiment on the impact of temperature during parasitoid nymphal development on interaction outcome (see results section 3.3.). N represents the number of parasitic tests (i.e. the number of dishes). The total number of larvae represents the number of larvae analyzed. For the parasitism rate, this is all larvae (alive and dissected); for the other parameters it is the number of mono-parasitized larvae only). The difference in the total number of larvae between the parasitism rate and the other parameters may be due to non-parasitized larvae but also to multi-parasitized larvae.

Host	Parasitoid	Parameter(s)	Temperature	N	Total number of larvae
D. melanogaster	ISm	Parasitism rate (Figure 4A)	20°C	8	230
			25°C	10	281
			30°C	12	341
		Parasitic success, host encapsulation capacity, escape from a capsule (Figure 4B)	20°C	8	183
			25°C	10	142
			30°C	8	113
D. melanogaster	ISy	Parasitism rate (Figure 4A)	20°C	13	371
			25°C	17	495
			30°C	18	524
		Parasitic success, host encapsulation capacity, escape from a capsule (Figure 4B)	20°C	13	283
			25°C	17	404
			30°C	9	58
D. yakuba	ISm	Parasitism rate (Figure 4A)	20°C	17	471
			25°C	16	457
			30°C	21	590
		Parasitic success, host encapsulation capacity, escape from a capsule (Figure 4B)	20°C	17	308
			25°C	16	273
			30°C	16	235
D. yakuba	ISy	Parasitism rate (Figure 4A)	20°C	5	134
			25°C	14	395
			30°C	11	314
		Parasitic success, host encapsulation capacity, escape from a capsule (Figure 4B)	20°C	5	83
			25°C	14	248
			30°C	0	0

Supplementary Table S4. Summary of pairwise contrasts comparing the effect of parasitoid rearing temperature on the outcome of the host-parasitoid interaction, reported in Figure 4. No statistical test was performed on the outcome of the ISm – *D. melanogaster* interaction because no variance in any outcome was observed. ISy females, reared at 30°C, did not parasitize *D. yakuba*. Thus, for this interaction, only two rearing temperature were compared (20°C vs 25°C).

Interaction	Parameter	Comparison	Estimate	Std. Error	Z value	Pr(>|Z|)
ISm - *D. yakuba*	Parasitism rate	20°C – 25°C	-1.55	0.58	-2.67	0.022 *
		20°C – 30°C	1.59	0.53	2.99	0.008 **
		25°C – 30°C	3.13	0.57	5.49	<0.001 ***
ISm - *D. melanogaster*	Parasitism rate	20°C – 25°C	-0.43	0.96	-0.45	0.89
		20°C – 30°C	3.24	0.91	3.56	0.001 **
		25°C – 30°C	3.68	0.87	4.25	<0.001 ***
ISy - *D. yakuba*	Parasitism rate	20°C – 25°C	-0.13	0.64	-0.20	0.84
ISy - *D. melanogaster*	Parasitism rate	20°C – 25°C	-0.37	0.63	-0.60	0.82
		20°C – 30°C	5.72	0.68	8.44	<0.001 ***
		25°C – 30°C	6.09	0.65	9.36	<0.001 ***
ISm - *D. yakuba*	Parasitism success	20°C – 25°C	-0.06	0.34	-0.17	0.98
		20°C – 30°C	4.23	0.45	9.45	<0.001 ***
		25°C – 30°C	4.29	0.45	9.45	<0.001 ***
ISm - *D. yakuba*	Parasitoid escape capacity	20°C – 25°C	-0.06	0.34	-0.17	0.98
		20°C – 30°C	4.23	0.45	9.45	<0.001 ***
		25°C – 30°C	4.29	0.45	9.45	<0.001 ***
ISy - *D. melanogaster*	Parasitism success	20°C – 25°C	0.49	0.16	3.09	0.006 **
		20°C – 30°C	3.83	0.73	5.25	<0.001 ***
		25°C – 30°C	3.34	0.73	4.60	<0.001 ***
ISy - *D. melanogaster*	Capsule inhibition	20°C – 25°C	0.71	0.34	2.07	0.096
		20°C – 30°C	2.93	1.10	2.68	0.020 *
		25°C – 30°C	2.22	1.09	2.03	0.10
ISy - *D. melanogaster*	Parasitoid escape capacity	20°C – 25°C	0.34	0.17	1.96	0.12
		20°C – 30°C	4.07	1.02	4.00	<0.001 ***
		25°C – 30°C	3.73	1.02	3.68	<0.001 ***
ISy - *D. yakuba*	Parasitism success	20°C – 25°C	-2.23	1.16	-1.92	0.056
ISy - *D. yakuba*	Capsule inhibition	20°C – 25°C	-0.42	0.63	-0.67	0.50
ISy - *D. yakuba*	Parasitoid escape capacity	20°C – 25°C	-3.04	1.57	-1.94	0.053

Supplementary Table S5. Sample size for the experiment on the temperature effect on the host capacity to encapsulate (see results section 3.2.). N represents the number of dishes. The total number of larvae represents the number of dissected larvae containing an oil drop.

Host	Temperature	N	Total number of larvae
D. melanogaster	20°C	13	229
	25°C	12	215
	30°C	13	212
D. yakuba	20°C	11	309
	25°C	11	241
	30°C	10	185

Supplementary Table S6. Summary of pairwise contrasts. Effect of temperature during larval development on the ability of *D. melanogaster* larvae to induce a melanized response to the oil drop, reported in Figure 3. No contrast was performed on *D. yakuba* since the main effect of temperature was not significant (p = X^2 = 2.8, p = 0.25).

species	Comparison	Estimate	Std. Error	Z value	Pr(>∣Z∣)
D. melanogaster	20°C – 25°C	-0.19	0.22	-0.85	0.67
	20°C – 30°C	2.39	0.44	5.39	<0.001 ***
	25°C – 30°C	2.57	0.44	5.83	<0.001 ***

Supplementary Table S7. Summary of pairwise contrasts. Impact of temperature during parasitoid nymphal development on the relative intensity of LbGAP, LbGAP2 and LbSPN in *L. boulardi* ISm venom and of LbSPN in *L. boulardi* ISy venom, reported in Figure 6.

Protein (parasitoid)	Comparison	Estimate	Std. Error	t ratio	Pr(>∣Z∣)	
LbGAP (ISm)	20°C – 25°C	0.001	0.004	0.20	0.98	
	20°C – 30°C	0.020	0.004	5.07	<0.001	***
	25°C – 30°C	0.019	0.004	4.87	0.001	**
LbGAP2 (ISm)	20°C – 25°C	-0.015	0.017	-0.86	0.67	
	20°C – 30°C	0.041	0.017	2.42	0.078	
	25°C – 30°C	0.056	0.017	3.28	0.017	*
LbSPN (ISm)	20°C – 25°C	-0.012	0.004	-3.14	0.022	*
	20°C – 30°C	0.009	0.004	2.31	0.092	
	25°C – 30°C	0.020	0.004	5.45	<0.001	***
LbSPN (ISy)	20°C – 25°C	-0.065	0.022	-2.90	0.033	*
	20°C – 30°C	0.081	0.022	3.65	0.009	**
	25°C – 30°C	0.146	0.022	6.55	<0.001	***

Supplementary Table S8. Summary of pairwise contrasts. Impact of temperature during parasitoid nymphal development on the total intensity of *L. boulardi* ISm and ISy bands reported in Figure 5.

Parasitoid line	Comparison	Estimate	Std. Error	t ratio	Pr(>|Z|)	
ISm	20°C – 25°C	-3.7e+5	3.2e+5	-1.16	0.50	
	20°C – 30°C	2.5e+5	3.2e+5	0.78	0.72	
	25°C – 30°C	6.2e+5	3.2e+5	1.94	0.17	
ISy	20°C – 25°C	7.9e+4	2.0e+5	0.40	0.92	
	20°C – 30°C	8.3e+5	2.0e+5	4.18	0.003	**
	25°C – 30°C	7.5e+5	2.0e+5	3.78	0.007	**

Supplementary Table S9. PERMANOVA for venom variation in each parasitoid line (*L. boulardi* ISm and ISy). Df: degrees of freedom. Sums of Sqs: sum of squares. F: F statistics. R2: partial R-squared. Pr(>F): p-value based on 5000 permutations.

Parasitoid	Variance Partition	Df	Sums Of Sqs	F	R^2	Pr(>F)
ISm	Temperature	2	9.04e+12	29.46	0.35	2e-04 ***
	Replicate	4	1.57e+12	2.56	0.06	1e-03 ***
	Residuals	98	1.50e+13		0.59	
	Total	104	2.57e+13		1.00	
ISy	Temperature	2	4.68e+12	25.08	0.32	2e-04 ***
	Replicate	4	8.84e+11	2.37	0.06	4e-04 ***
	Residuals	98	9.15e+12		0.62	
	Total	104	1.47e+13		1.00	

Supplementary Table S10. Correlation values of *L. boulardi* ISm bands to linear regression (arrow from LDA) before and after partial correlation analysis. Cluster numbers are from the clustering analysis (see Supplementary Figure S2). Protein bands in bold are those whose intensity varies with temperature. A "↗" indicates an increase in band intensity with increasing temperature while a "↘" indicates a decrease with increasing temperature. To be considered a changing protein band, the sign (+ or –) of the correlation must be the same before and after the partial correlation analysis and the significance level must be less than 0.05 before and after the partial correlations. Partial correlations were performed on clusters with at least two protein bands significantly correlated with the arrow.

Band	Cluster	Before partial correlations		After partial correlations		Change with increasing temperature
		Correlation	p-value	Correlation	p-value	
**3**	-	**-0.33**	**0.018**			↗
**6**	-	**0.36**	**0.006**			↘
8	-	0.29	0.093			
**10**	-	**0.41**	**<0.001**			↘
12	-	-0.22	0.785			
20	-	0.17	1.000			
21	-	0.20	1.000			
**27**	1	**0.52**	**<0.001**	**0.38**	**0.002**	↘
28	1	**0.47**	**<0.001**	0.26	0.186	
32	1	0.15	1.000	-0.18	1.000	
33	1	0.14	1.000	-0.01	1.000	
15	2	-0.13	1.000	0.17	1.000	
**23**	2	**0.65**	**<0.001**	**0.74**	**<0.001**	↘
24	2	0.26	0.226	-0.55	<0.001	
25	2	**0.34**	**0.011**	0.12	1.000	
4	3	**0.47**	**<0.001**	0.11	1.000	
**5**	3	**0.59**	**<0.001**	**0.41**	**<0.001**	↘
1	4	**-0.62**	**<0.001**	0.35	0.010	
**2**	4	**-0.90**	**<0.001**	**-0.63**	**<0.001**	↗
7	4	**-0.56**	**<0.001**	0.11	1.000	
9	4	**0.74**	**<0.001**	0.22	0.576	
11	4	**0.73**	**<0.001**	0.26	0.218	
16	4	**-0.73**	**<0.001**	-0.10	1.000	
17	4	**-0.81**	**<0.001**	-0.28	0.121	
18	4	**-0.66**	**<0.001**	0.21	0.754	
**22**	4	**0.80**	**<0.001**	**0.34**	**0.016**	↘
19	5	0.00	1.000			
26	5	0.00	1.000			
29	5	-0.18	1.000			
13	6	**0.40**	**0.001**	0.18	1.000	
**14**	6	**0.49**	**<0.001**	**0.34**	**0.007**	↘
**30**	7	**-0.32**	**0.029**			↗
31	7	-0.15	1.000			
34	8	-0.27	0.217			
**35**	8	**-0.68**	**<0.001**			↗

Supplementary Table S11. Correlation values of *L. boulardi* ISy bands to linear regression (arrow from LDA) before and after partial correlation analysis. Cluster numbers are from the clustering analysis (see Supplementary Figure S2). Protein bands in bold are those whose intensity varies with temperature. A "↗" indicates an increase in band intensity with increasing temperature while a "↘" indicates a decrease with increasing temperature. To be considered a changing protein band, the sign (+ or –) of the correlation must be the same before and after the partial correlation analysis and the significance level must be less than 0.05 before and after the partial correlations. Partial correlations were performed on clusters with at least two protein bands significantly correlated with the arrow.

Band	Cluster	Before partial correlations		After partial correlations		Change with increasing temperature
		Correlation	p-value	Correlation	p-value	
**1**	-	**-0.33**	**0.019**			↗
3	-	0.18	1.000			
5	-	0.13	1.000			
**11**	-	**0.40**	**0.001**			↘
**18**	-	**0.44**	**<0.001**			↘
**24**	-	**-0.42**	**<0.001**			↗
30	-	-0.10	1.000			
6	1	**-0.59**	**<0.001**	-0.28	0.054	
17	1	**0.49**	**<0.001**	0.15	1.000	
**19**	1	**-0.66**	**<0.001**	**-0.43**	**<0.001**	↗
25	1	**0.44**	**<0.001**	0.01	1.000	
4	2	**0.63**	**<0.001**	0.18	0.927	
12	2	**0.66**	**<0.001**	0.26	0.134	
15	2	**0.68**	**<0.001**	-0.04	1.000	
**16**	2	**0.77**	**<0.001**	**0.50**	**<0.001**	↘
23	2	**-0.70**	**<0.001**	-0.28	0.061	
9	3	**-0.57**	**<0.001**	0.23	0.281	
**10**	3	**-0.88**	**<0.001**	**-0.79**	**<0.001**	↗
20	3	**-0.54**	**<0.001**	-0.08	1.000	
13	4	0.03	1.000			
26	4	0.20	1.000			
27	4	0.09	1.000			
**28**	4	**0.49**	**<0.001**			↘
29	4	-0.12	1.000			
2	5	-0.16	1.000			
14	5	-0.23	0.614			
7	6	-0.11	1.000			
8	6	0.24	0.455			
**21**	7	**-0.34**	**0.013**			↗
22	7	0.13	1.000			
31	8	**0.37**	**0.003**	0.14	1.000	
32	8	**0.39**	**0.001**	0.19	0.705	
